# Atypical Variant of Posterior Reversible Encephalopathy Syndrome in the Setting of Renovascular Hypertension: Case Report and Review of Literature

**DOI:** 10.7759/cureus.3573

**Published:** 2018-11-12

**Authors:** Carli Wittgrove, Harleen Kaur, Junaid H Siddiqui

**Affiliations:** 1 Neurology, University of Missouri, Columbia, USA; 2 Neurology, Univeristy of Missouri, Columbia, USA

**Keywords:** posterior reversible encephalopathy syndrome, brainstem, hypertension, renal artery stenosis, pres

## Abstract

Posterior reversible encephalopathy syndrome (PRES) is a clinico-radiologic syndrome resulting in subcortical vasogenic edema appreciated on T2/fluid-attenuated inversion recovery (FLAIR) sequence of magnetic resonance imaging (MRI). PRES classically involves bilateral parieto-occipital lobes and is usually reversible. Atypical variant of PRES includes the involvement of brainstem, basal ganglia, thalami, or periventricular white matter. We report an unusual case of PRES with isolated brainstem involvement with periventricular white matter changes in a patient with renovascular hypertension from unilateral renal artery stenosis. To our knowledge, this is the first case of secondary hypertension from renal artery stenosis resulting in the atypical variant of PRES.

## Introduction

Posterior reversible encephalopathy syndrome (PRES) was first defined by Hinchey and his colleagues in 1996 on a series of 15 patients. PRES includes a myriad of neurological symptoms ranging from headache, loss of consciousness, visual disturbances, seizures and focal neurological deficits [1]. The most common radiologic presentation of PRES results in vasogenic edema appreciated on T2 and fluid-attenuated inversion recovery (FLAIR) magnetic resonance imaging (MRI) images mainly involving the bilateral parieto-occipital region and are usually reversible [2]. The atypical presentation of PRES may involve the brainstem, basal ganglia, thalami and periventricular white matter [3]. We report an unusual case of PRES with isolated brainstem involvement and periventricular white matter changes in the setting of severe renovascular hypertension from right-sided renal artery stenosis.

## Case presentation

**Figure 1:**
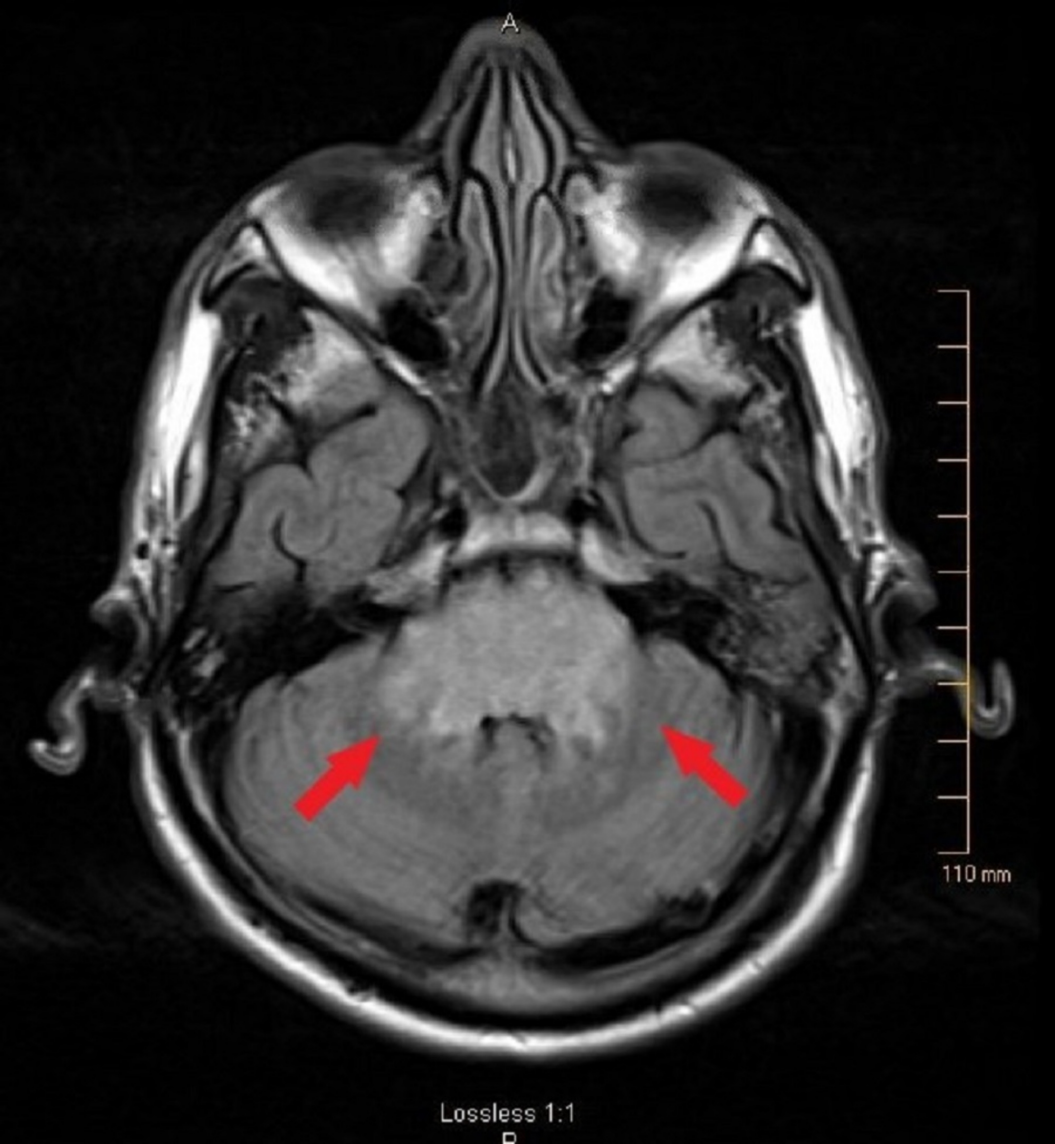
MRI on admission shows hyperintense signals on FLAIR sequence involving the brainstem and extending in the cerebellar peduncles bilaterally (red arrows). MRI: Magnetic resonance imaging; FLAIR: Fluid-attenuated inversion recovery.

**Figure 2:**
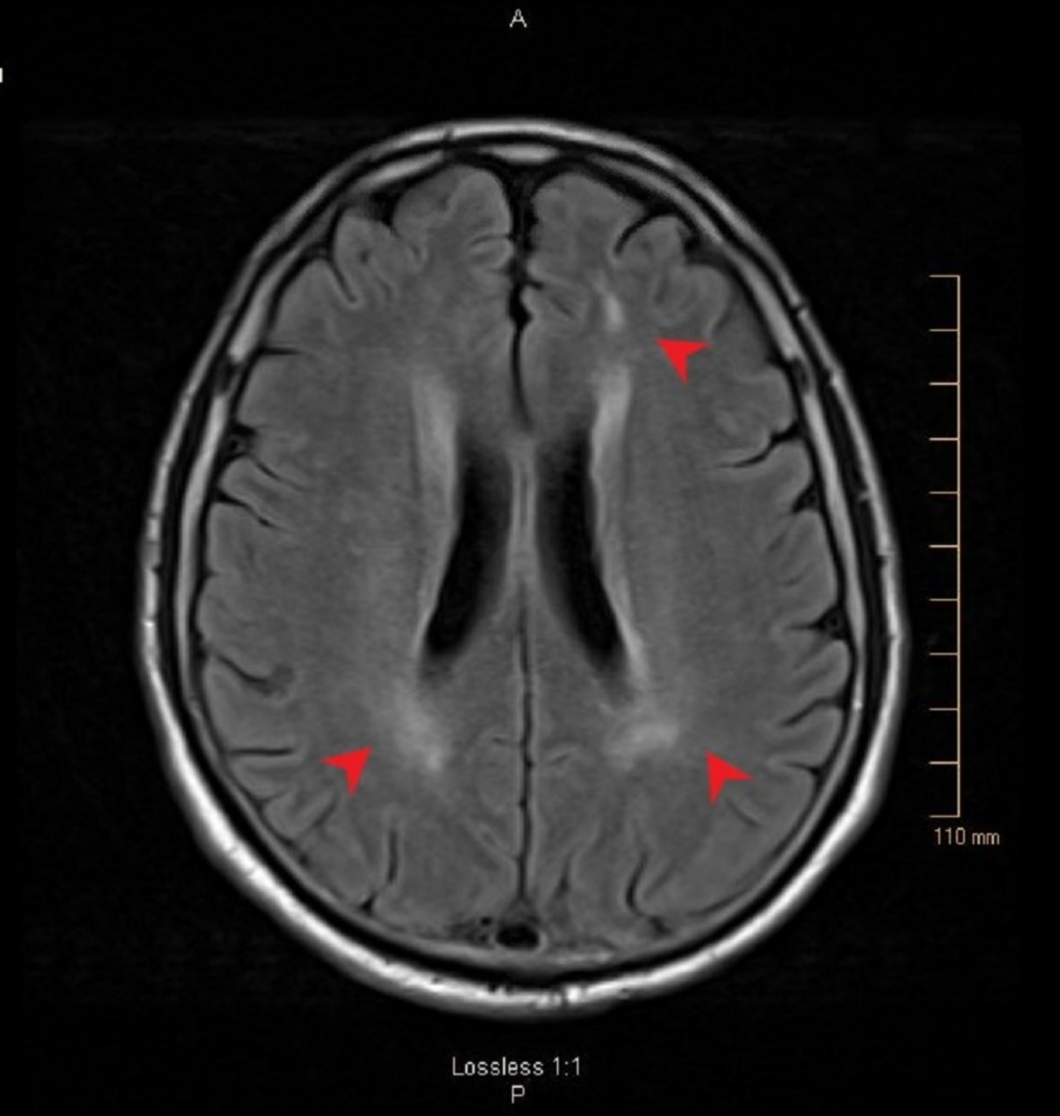
MRI on admission shows periventricular white matter changes on FLAIR sequence (red arrowhead). MRI: Magnetic resonance imaging; FLAIR: Fluid-attenuated inversion recovery.

A 57-year-old, otherwise healthy male construction worker, presented to the emergency room (ER) after a mechanical fall from tripping and hitting his occiput on a flower pot. This resulted in reported loss of consciousness for around 10 minutes. He complained of confusion, right-sided weakness, double vision and difficulty in speaking, upon recovering from the fall. On exam, the patient was alert, oriented to time, place and person but had dysarthria, ataxia, nystagmus, right-sided weakness and hyperreflexia (with positive Hoffman’s bilaterally, and up-going plantar response on the left side). The family denied any seizure-like activity during this time or in past. His systolic blood pressure (SBP) in the ER ranged from 202 to 220 mmHg. The initial computed tomography (CT) scan was negative for acute intracranial findings. The MRI scan suggested non-enhancing T2/FLAIR hyperintensities involving the brainstem and extending in the cerebellar peduncles bilaterally (Figure 1). T2/FLAIR hyperintensities were also noted in periventricular white matter area (Figure 2). The MRI of the cervical spine did not show any herniation of the cerebellar tonsils down the foramen magnum. However, it did suggest some degenerative changes in cervical spine at C5-6 level.

The patient was transferred to the neuro-intensive care unit (NICU) for further management of high blood pressure that included carvedilol 12.5 mg three times a day, labetalol (as needed) with a goal of SBP between 140 and 180 mmHg.

The lumbar puncture showed elevated proteins of 80 mg/dl (normal range: 15–60 mg/dl), and normal white blood cell count (WBC) of 4/mcL (microliter), red blood cell count (RBC) of 17/mcL and glucose of 63 mg/dl with an opening pressure of 13 cm water. All extensive workup for infective and neoplastic causes of brainstem white matter changes was negative for cytology, acid-fast bacilli (AFB), cryptococcus, human immunodeficiency virus (HIV), herpes simplex virus (HSV), venereal disease research laboratory test (VDRL), and West Nile Virus. The levels of vitamin B1, B2, B12, D, E were also within normal limits.

Our patient’s past medical history included ulcerative colitis, hemorrhoids, and colonic polyps. His family history was positive for hypertension, diabetes, and cancer. He denied any alcohol use, but admitted to smoking a pack of cigarette daily. He denied any illegal drug use other than occasional use of marijuana.

**Figure 3:**
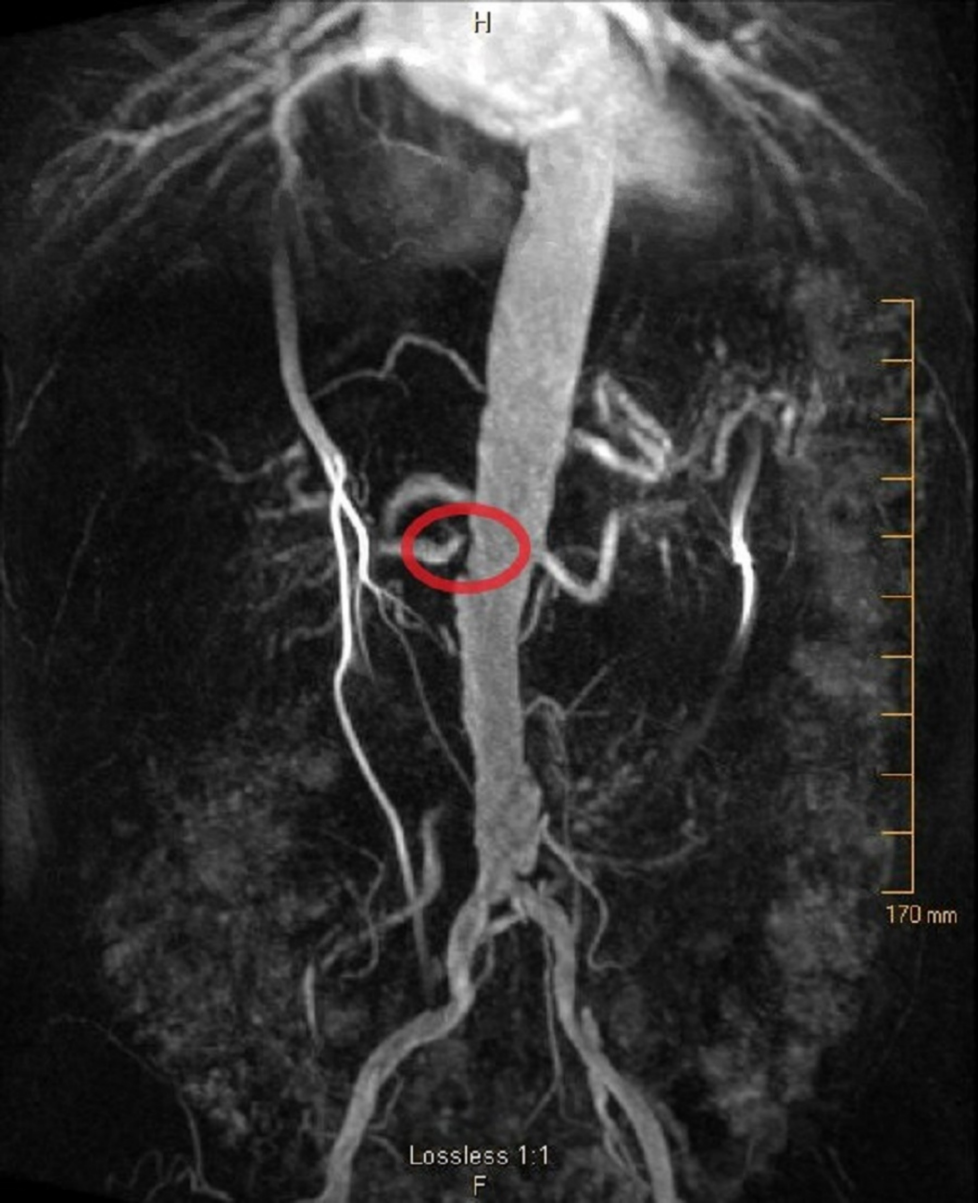
Severe renal artery stenosis on the right side confirmed by MRA (encircled in red). MRA: Magnetic resonance angiogram.

Despite aggressive attempts at management of blood pressure, it was refractory to labetalol, spironolactone, nifedipine, and hydralazine. This led to further evaluation of secondary causes of hypertension including renal artery ultrasound and aldosterone to renin ratio. The aldosterone to renin ratio was within normal range but the renal artery ultrasound revealed right renal artery stenosis which was further confirmed by magnetic resonance angiogram (MRA) (Figure 3).

**Figure 4:**
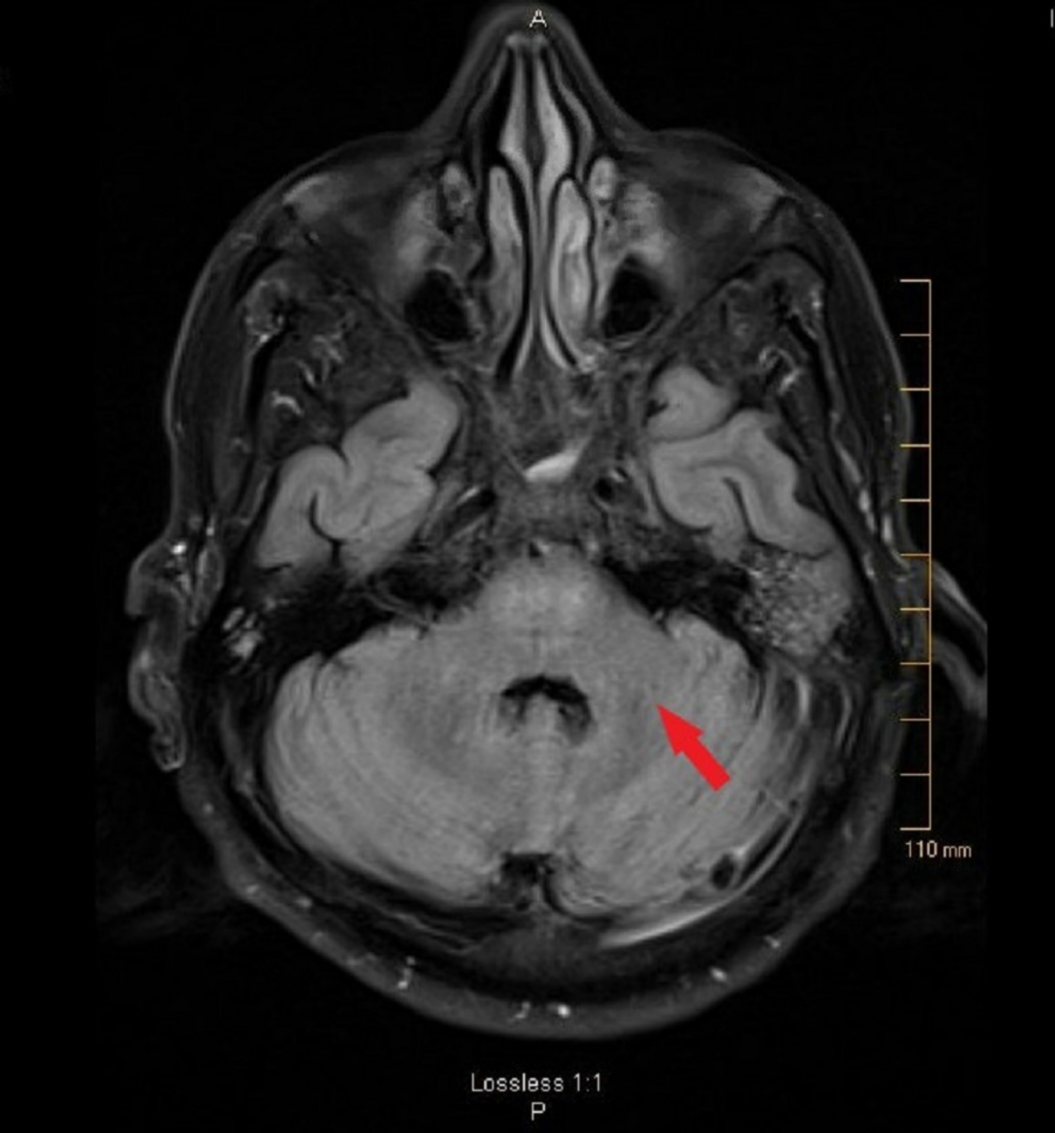
Follow-up MRI shows marked regression of lesions involving the brainstem and cerebellar peduncles on FLAIR sequence (red arrow). MRI: Magnetic resonance imaging; FLAIR: Fluid-attenuated inversion recovery.

Right-sided renal artery stenting was performed by interventional radiology (IR). Post-stenting, his blood pressure returned to normal range. At this time his physical exam was significant for dysarthria and hyperreflexia without any right-sided weakness. He was evaluated by physical therapy/occupational therapy (PT/OT) and discharged to a rehabilitation center. Subsequently, on follow-up in the clinic, a repeat examination showed significant resolution of his dysarthria and improvement in pyramidal and cerebellar signs. The MRI of the brain, during this time, showed marked improvement in T2/FLAIR hyperintensities in the brainstem and cerebellar peduncles (Figure 4).

## Discussion

PRES is a clinico-radiologic syndrome characterized by reversible subcortical vasogenic edema seen on neuroimaging predominantly involving the parieto-occipital lobes bilaterally. Radiologically, the vasogenic edema can be best appreciated as hyperintense signals on T2/FLAIR sequences of MRI [2]. Clinically, PRES can manifest with headache, altered consciousness, papilledema, visual disturbances, seizures and neurological deficits [4]. Most patients recover clinically and radiologically within a few weeks to months of the symptoms. PRES can occur in the setting of hypertensive emergencies, eclampsia, immune-suppressive/cytotoxic drugs, chronic renal disease, liver failure, bone marrow transplant, autonomic instability, and endocrine dysfunction [5].

The pathophysiology underlying this syndrome is explained by two mechanisms, endothelial dysfunction and failure of auto-regulation. The hypertensive episodes result in cerebral vasoconstriction which leads to cerebral hyper-perfusion and vascular leakage. Further, it is also postulated that dense sympathetic innervation in the anterior circulation relatively protects the neuronal tissue from blood pressure fluctuations as compared to posterior circulation which has relatively sparse sympathetic innervation [6]. However, 30% of patients with PRES have normal or moderate rise in blood pressure which cannot be explained by the hyper perfusion theory [7]. The second theory explains endothelial dysfunction as the possible trigger for PRES. The endogenous (eclampsia, sepsis) and exogenous toxins (immune suppressive agents) cause the release of pro-inflammatory mediators and vasoactive substances which result in vascular leakage, endothelial dysfunction and vasogenic edema [8].

Atypical variants of PRES involving the brainstem, basal ganglia, cerebellum, and deep white matter changes have also been documented in the literature [9-11]. In 2007, McKinney et al. documented the incidence of atypical variant in 76 patients with PRES. They noted 98.7% had involvement of parieto-occipital regions, 78.9% involved posterior frontal regions, 68.4% involved temporal, 30.3% had involvement of thalamus, 34.2% involved the cerebellum, 18.4% involved the brainstem and 11.8% involved the basal ganglia [3]. In 2012, McKinney et al. published another study highlighting the involvement of brainstem in 17.7% of 124 patients with PRES and 18.5% patients had involvement of basal ganglia [9]. However, the exact pathophysiology behind the involvement of the atypical variant is not clearly understood. The best possible explanation given in the literature postulates that severe hypertension and increased perfusion pressure results in excessive fluid leakage in the brainstem along with ‘sparing’ of the distal vertebrobasilar system and development of collateral sympathetic innervation, which has a protective effect on the occipito-parietal areas through the posterior communicating artery [12].

Our case presented as atypical variant of PRES. The MRI showed hyperintense signals in the brainstem mainly involving the pons and bilateral cerebellar peduncles on T2/FLAIR sequences without involvement of the parieto-occipital lobes (Figure 1). All other possible causes of brainstem white matter changes were also ruled out. Our patient developed renovascular hypertension secondary to right-sided renal artery stenosis which resulted in brainstem hypertensive encephalopathy. Our case fulfilled the two main features for brainstem hypertensive encephalopathy. Firstly, the clinical symptoms were comparatively mild in comparison with the neuroimaging abnormality seen on the MRI. This typical clinico-radiologic dissociation is seen in hypertensive encephalopathy involving the brainstem [13]. Secondly, subsequent regression of the lesion was noted on neuroimaging after effective control of blood pressure [14].

Our case is noteworthy because it is the only case of PRES with diffuse brainstem involvement in the setting of renovascular hypertension. To our knowledge, this is the only case of severe secondary hypertension resulting in the atypical variant of PRES in the setting of unilateral renal artery stenosis.

## Conclusions

It is not uncommon to see the atypical variant of PRES in the healthcare setting. Hypertensive encephalopathy can occur in the setting of renovascular hypertension and precipitate as atypical variant of PRES. Early diagnosis and effective management of blood pressure can help to further prevent adverse neurological sequelae of epilepsy, persistent neurological deficits, cerebral ischemia, and infarction.
